# Age-Dependent Lethality in Ducks Caused by Highly Pathogenic H5N6 Avian Influenza Virus

**DOI:** 10.3390/v12060591

**Published:** 2020-05-29

**Authors:** Yunyueng Jang, Sang Heui Seo

**Affiliations:** 1 Laboratory of Influenza Research and College of Veterinary Medicine, Chungnam National University, Daejeon 34134, Korea; jyy1915@gmail.com; 2 Institute of Influenza Virus, Chungnam National University, Daejeon 34134, Korea

**Keywords:** avian influenza virus, H5N6, age-dependent lethality, ducks

## Abstract

Ducks show notably higher resistance to highly pathogenic avian influenza viruses as compared to chickens. Here, we studied the age-dependent susceptibility in ducks to the infections caused by highly pathogenic avian influenza viruses. We intranasally infected ducks aged 1, 2, 4, and 8 weeks with highly pathogenic H5N6 avian influenza viruses isolated in South Korea in 2016. All the 1-and 2-week-old ducks died after infection, 20% of 3-week-old ducks died, and from the ducks aged 4 and 8 weeks, all of them survived. We performed microarray analysis and quantitative real-time PCR using total RNA isolated from the lungs of infected 2- and 4-week-old ducks to determine the mechanism underlying the age-dependent susceptibility to highly pathogenic avian influenza virus. Limited genes were found to be differentially expressed between the lungs of 2- and 4-week-old ducks. Cell damage-related genes, such as CIDEA and ND2, and the immune response-related gene NR4A3 were notably induced in the lungs of infected 2-week-old ducks compared to those in the lungs of infected 4-week-old ducks.

## 1. Introduction

Influenza viruses are RNA viruses and belong to the family Orthomyxoviridae [1,2]. Influenza viruses are usually classified as A, B, C, or D based on serological reactivity to the nucleoprotein and matrix protein [1,3]. Among them, influenza A viruses are genetically variant and have a broad spectrum of host ranges [1,2]. Influenza A viruses are further divided into subtypes based on hemagglutinin (HA) and neuraminidase (NA). Until now, 16 HA and 9 NA types are found to be circulating in birds [2,4].

A highly pathogenic (HP) avian influenza virus, H5N1, infected 18 people in 1997, of which 6 of them died [5]. This infection was related to the A/goose/Guangdong/1/1996 infection (H5N1) [6]. Since 2003, highly pathogenic H5N1 avian influenza viruses have spread to poultry around the world [7,8,9,10,11,12].

HP H5N1 viruses have undergone genetic drift and reassortment to diversify into 10 different clades [13]. In 2008, HP H5 viruses in clade 2.3.4 reassorted with N2, N5, N6, and N8 genes from avian influenza viruses in China to cause a widespread infection [14]. In fact, the outbreaks of H5N6 and H5N8 have occurred in many countries, such as China, Korea, Japan, Taiwan, Canada, Germany, Poland, the United States, and the United Kingdom [15,16,17,18,19,20,21,22,23,24,25,26,27].

It is known that the HP avian influenza virus causes more severe disease in chicken than in ducks [28]. Ducks can maintain t H5 virus in their bodies without showing any severe symptoms and become sources of virus spread to susceptible chickens [29]. Chicken infected with highly pathogenic H5Nx viruses suffer from systemic infection, resulting in multiple organ failure, damage to cardiovascular and nervous systems, and, ultimately, death [30].

In this study, we assess the age-dependent susceptibility to HP H5N6 in duck and further investigate the mechanism for the susceptibility.

## 2. Materials and Methods

### 2.1. Viruses and Animals

Highly pathogenic (HP) avian influenza viruses, A/Waterfowl/Korea/S57/2016 (H5N6) (clade 2.3.4.4.), were grown in fertilized 10-day-old chicken eggs. The fertilized Pekin duck eggs were obtained from the local farm in South Korea and were incubated and hatched in our laboratory.

Animal experiments were done in the animal BSL-3 facility approved by the Korean government. Animal Rights Statement: Chungnam National University (CNU) Internal Animal Use Committee approved the protocol (CNU00868, 01.04.2017) for duck infection studies.

### 2.2. Infection of Ducks with Highly Pathogenic Avian Influenza Viruses

Ducks aged from 1 to 8 weeks (*n* = 10 per group) were intranasally (i.n.) infected with 0.25 mL of 10^6^ egg infectious dose 50/mL (EID50/mL) of A/Waterfowl/Korea/S57/2016 (H5N6) (clade 2.3.4.4.). Mortality was observed for 14 days post infections (p.i.).

### 2.3. Measurement of Virus Titer in the Lungs of Ducks

Ducks aged 2 or 4 weeks old were intranasally infected with 0.25 mL of 10^6^ EID_50_/_mL_ of A/Waterfowl/Korea/S57/2016 (clade 2.3.4.4.). On day 3 p.i., the surviving ducks (*n* = 3 per group) were euthanized with T61 (Intervet, Canada). Lung tissues were collected in 10% suspension in PBS (pH 7.4) supplemented with 1× antibiotic antimycotic solution (Sigma-Aldrich, St. Louis, MO, USA) and homogenized with BeadBlaster 24 (Benchmark, NJ, USA). The homogenized samples were centrifuged for 3 min at 13,000 rpm, and the supernatants were used for viral titers in plaque-forming units using MDCK cells. We selected a 3-day p.i. time point to euthanize the ducks as most of the ducks that were less than 3 weeks old had already died.

### 2.4. Histopathology of Duck Lungs

The parts of lung tissues from ducks used for viral titration in lungs were submerged in 10% neutral-buffered formalin before embedding in paraffin. Thin 5-µm sections were cut and later stained with hematoxylin and eosin (H&E). The stained tissues were observed under an Olympus DP70 microscope (Olympus Corporation, Tokyo, Japan).

### 2.5. Analysis of Gene Expression in the Lungs of Ducks Using DNA Microarray

One gram of lung tissues from ducks used for viral titration was pulverized in liquid nitrogen using sterile mortars and pestles. The ground tissue samples were taken into a cold 2-mL microtube, and then 1000 μL of TRIzol (Invitrogen, Waltham, MA, USA) was added. The TRIzol solution with tissues was centrifuged at 12,000× *g* for 10 min at 4 °C and then the supernatant phase was collected into a new 2-mL tube and incubated on ice for 5 min. Chloroform (200 μL) (Sigma-Aldrich, St. Louis, MO, USA) was added to the samples and the sample mixture was centrifuged at 12,000× *g* for 15 min at 4 °C. The aqueous phase was harvested, was added with the chilled 500-μL isopropyl alcohol (Sigma-Aldrich, St. Louis, MO, USA), and was incubated on ice for 10 min before the solution was centrifuged at 12,000× *g* for 10 min at 4 °C. The supernatant was decanted; the pellet was washed with 1000 μL of 75% chilled ethanol (Sigma-Aldrich, USA) and centrifuged at 9000× *g* for 5 min at 4 °C. The RNA pellet was recovered after the supernatant was decanted, and was air-dried at room temperature. RNA was dissolved in 50 μL of DEPC-treated water and treated with DNase (Qiagen, Venlo, The Netherlands). The purified RNA was sent out for Microarray analysis (Ebiogene, Seoul, South Korea) to analyze the gene expression. A chicken DNA chip (Affymetrix Chicken 1.0 ST array) was used for the microarray analysis of total duck RNA. We used the chicken DNA chip as described [31] as no duck DNA chip was available.

### 2.6. Quantification of Duck Genes by Real-Time PCR

Genes expressed over 2-fold in the infected 2-week-old ducks compared to those in the infected 4-week-old ducks in microarray analysis were quantified by quantitative real-time PCR. Then, 1 μL of oligo dT primers (0.5 pmol) (Promega, Madison, WI, USA) was added to 9 μL total duck RNA (4 μg) to prepare the cDNA. The reaction mixture was run for 5 min at 70 °C and then incubated for 5 min at 4 °C. This mixture was added with 4 μL of 25 mM MgCl_2_, 4 μL of 5× reverse transcriptase enzyme buffer, 1 μL of RNase inhibitor, 1 μL of reverse transcriptase, and 1 μl of dNTP (10 mM) and was incubated for 5 min at 25 °C, 60 min at 42 °C, and 15 min at 70 °C. According to the manufacturer’s protocol, quantification real-time PCR was carried out using Roto-Gene 6000 apparatus (Corbett, Mortlake, Australia) and SensiMix Plus SYBR (Quantace, London, UK). The reactions were run in duplicates and contained total final reaction volume of 20 μL: 1 μL of cDNA, 10 μL of SYBR mixture, duck gene-specific primers (1 μL of forward primer (20 pmol) and 1 μL of reverse primer (20 pmol)) (Table 1), and 7 μL of water with real-time PCR conditions—40 cycles of 5 s at 95 °C, 15 s at 60 °C, and 25 s at 72 °C. Gene expression levels were normalized to those of duck glyceraldehyde-3-phosphate dehydrogenase (GAPDH). Results were quantified using the delta–delta CT method. ExDEGA Graphic Plus v2.0 was used for gene analysis.

### 2.7. Measurement of Viral Titers by Plaque Forming Units (P.F.U.)

The supernatants of homogenized lung tissues were serially diluted 10-fold and were infected into MDCK cells in the 6-well plates. The infected cells were overlayed with 2% electrophoretic-grade agar (LPS solution, Daejeon, South Korea), and, 4 days later, 2% neutral red was added. Plaques were counted as described [32].

### 2.8. Statistical Analysis

The statistical significance was analyzed by the Mann–Whitney U test. *P*-value of less than 0.05 (*P* < 0.05) was considered significant.

## 3. Results

**Figure 1:**
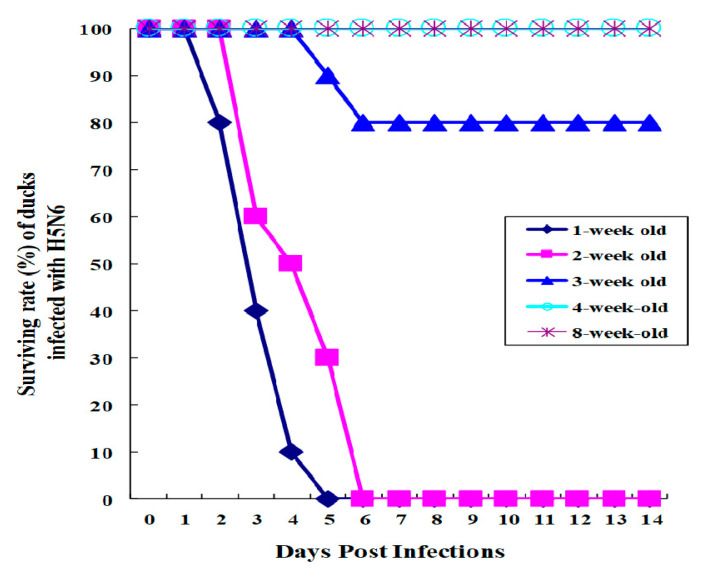
Surviving rate of ducks infected with HP H5N6 avian influenza virus. Ducks aged from 1, 2, 4, or 8 weeks old (*n* = 10 per group) were i.n. infected with 0.25 mL of 10^6^EID_50_/_mL_ of A/Waterfowl/Korea/S57/2016 (H5N6) (clade 2.3.4.4.). Duck surviving rate was monitored for 14 days p.i.

Age-dependent susceptibility in ducks against HP H5N6 avian influenza virus is not yet known though there were reports on age-dependent susceptibility of ducks against the HP H5N1 avian influenza virus [33,34,35]. To assess the age-dependent mortality, we intranasally (i.n.) inoculated ducks (*n* = 10 per group) aged 1 to 8 weeks with HP avian influenza A/Waterfowl/Korea/S57/2016 (H5N6) (clade 2.3.4.4.). All ducks aged between 4 and 8 weeks infected with HP H5N1 viruses survived (Figure 1). All ducks aged 1 and 2 weeks old that were infected with HP H5N6 viruses died within 6 days post-infection (p.i.), while from the infected ducks aged 3 weeks old, 20% of them died (Figure 1). All ducks aged 4 and 8 weeks old survived. Results suggest that ducks gain resistance to HP H5N6 avian influenza virus after the age of 3 weeks. All PBS-mock infected ducks survived (data not shown). All inoculated ducks were found to be infected, as observed from the swabbed samples in cloacae of ducks inoculated into MDCK cells showed cytopathic effects (data not shown).

**Figure 2:**
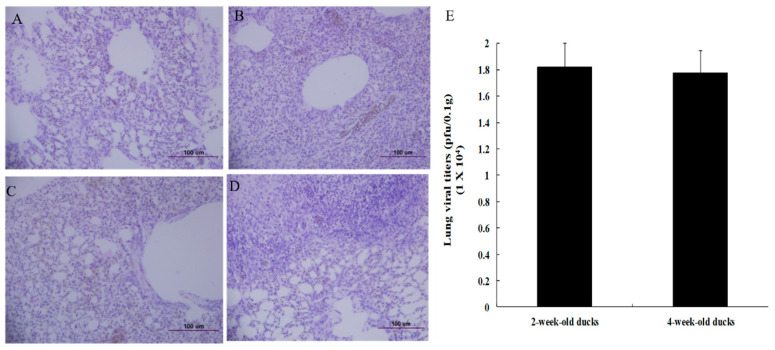
Viral titers and histopathology in the lungs of ducks infected with HP H5N6 avian influenza virus. Lung tissues from ducks used for viral titration were stained with H&E. (**A**) The lung tissue of uninfected 2-week-old duck, (**B**) the lung tissue of infected 2-week-old duck, (**C**) the lung tissue of uninfected 4-week-old duck, (**D**) The lung tissue of infected 4-week-old duck, (**E**) Viral titers in lungs of ducks. Ducks aged 2 or 4 weeks old were i.n. infected with 10^6^ EID_50_/_mL_ of A/Waterfowl/Korea/S57/2016 (clade 2.3.4.4.). The survived ducks (*n* = 3 per group) were euthanized on day 3 p.i. and lung tissues were collected. The viral titers were determined by p.f.u. using MDCK cells.

We further wanted to estimate the difference in the pathological damage to the lung tissues between the 2- and 4-week-old infected ducks. Upon examining, the lungs of the infected duck aged 2 weeks (Figure 2B) showed more severe interstitial pneumonia than that of the 4-week-old infected duck (Figure 2D). We then measured the viral titers in the lung tissues of the 2- and 4-week-old ducks and observed that the viral titers were similar (Figure 2E). The mean titer in the lungs of infected ducks aged 2 weeks old was 1.82 × 10^4^ pfu/0.1 g and that in the 4-week-old duck was 1.77 × 10^4^ pfu/0.1 g.

**Figure 3:**
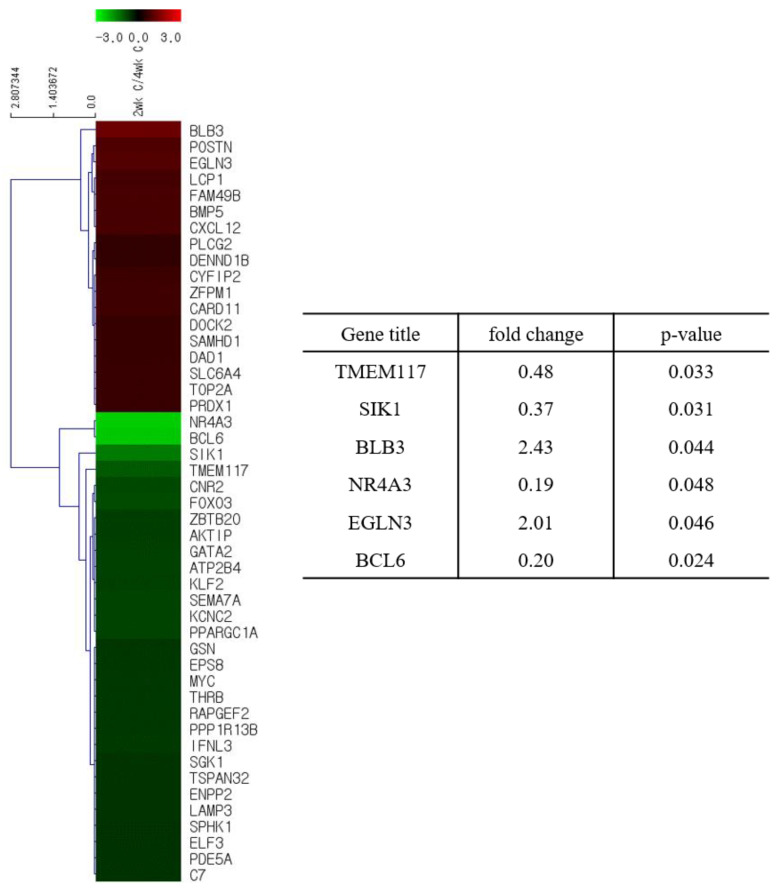
Heat maps and the fold change of differentially expressed genes in the lung of PBS-mock infected ducks. Total RNA was collected from one gram of lung tissue from ducks. The purified RNA was used for microarray using a chicken DNA chip: The differentially expressed genes in the lungs of a 2-week-old duck compared to those in the lungs of a 4-week-old duck.

**Figure 4:**
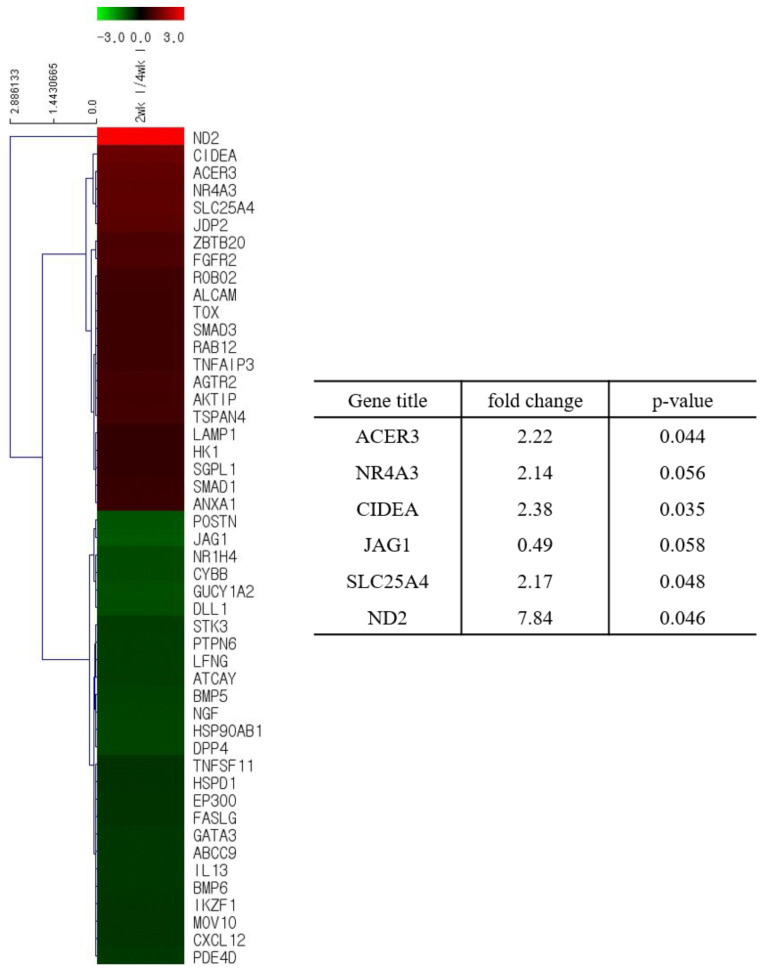
Heat maps and the fold change of differentially expressed genes in the lung of infected ducks. Total RNA was collected from one gram of lung tissue from ducks used for viral titration. The purified RNA was used for microarray using a chicken DNA chip: The differentially expressed genes in the lungs of a 2-week-old duck compared to those in the lungs of a 4-week-old duck.

We analyzed the total gene expression in the lungs of the ducks to explain the age-dependent resistance of ducks to HP avian influenza virus. We collected total RNA from the lungs of 2- and 4 week-old infected or PBS-mock infected ducks and performed microarray analysis. Among 1491 genes related to anti-viral activity, cell damage, immune responses, and inflammatory responses, 38 and 74 genes were found to be significantly modulated in the lungs of PBS-mock infected and infected ducks, respectively (Figure 3 and Figure 4, Table S1). Among 38 genes modulated in the lungs of PBS-mock infected ducks, two genes, BLB3 (major histocompatibility complex class II beta chain BLB3) and EGLN3 (egl-9 family hypoxia-inducible factor 3) were modulated more than 2-fold (Figure 3 and Figures S1–S4). This result suggests that age affects the gene expression in ducks, resulting in a difference in disease pathogenesis. When we analyzed genes related to the anti-viral pathways in the lungs of infected ducks, there was no gene that was expressed over 2-fold of difference between the lungs of 2- and 4-week-old ducks (Figure 4 and Figure S5). There were three cell-damage related genes, CIDEA (cell death-inducing DFFA-like effector a), ND2 (NADH dehydrogenase subunit 2), and SLC25A4 (solute carrier family 25, member 4), which were expressed over 2-fold in the lungs of infected 2-week-old ducks compared to those in the lungs of infected 4-week-old ducks (Figure 4 and Figure S6). In addition, there was one gene, NR4AS (nuclear receptor subfamily 4, group A, member 3), related to immune responses, which was expressed over 2-fold in the lungs of infected 2-week-old ducks than in the 4-week-old ducks (Figure 4 and Figure S7). Concerning the genes related to inflammatory response genes, one gene, ACER3 (alkaline ceramidase 3), was expressed over 2-fold in the lungs of 2-week-old ducks than in the lungs of 4-week-old ducks (Figure 4 and Figure S8). When we compared the gene expression between infected duck lungs and the PBS-mock infected duck lungs, among 1491 genes related to anti-viral activity, cell damage, immune responses, and inflammatory responses, 25 genes, including ND2, were modulated over 2-fold in the infected lungs of 2-week-old ducks (Figure S9), while 37 genes, including POSTN (perisostin, osteoblast specific factor), were modulated over 2-fold in the infected lungs of 4-week-old ducks (Figure S10).

**Figure 5:**
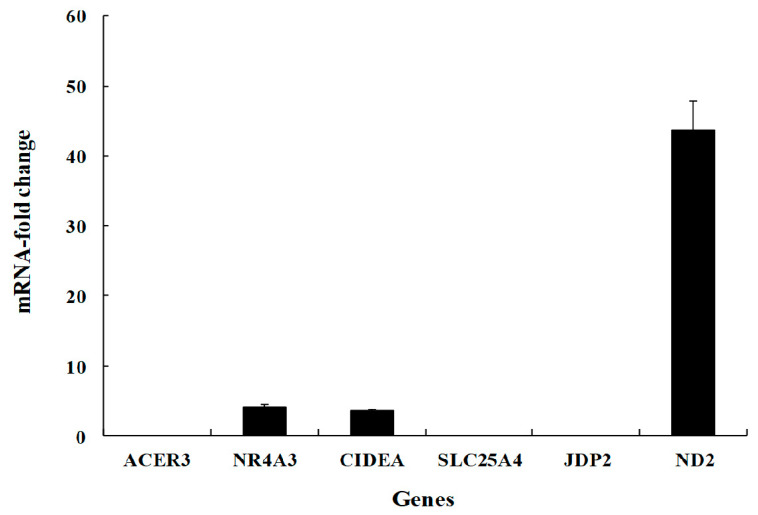
Quantitative real-ti me PCR of differentially expressed genes. Genes expressed over 2-fold in the infected 2-week-old ducks compared to those in the infected 4-week-old ducks using oligo dT primers and SYBR die. * *p* < 0.05; ** *p* < 0.001.

We performed the quantitative real-time PCR in mRNA genes expressed over 2-fold in the lungs of 2-week-old ducks and in the lungs of 4-week-old ducks. The fold increase of NR4AS, CIDEA, and ND2 genes in the lungs of infected 2-week-old ducks than in the lungs of infected 4-week-old ducks were 4.1, 3.5, and 43.7, respectively (Figure 5).

## 4. Discussion

The recent Asian-origin HP H5NX avian influenza viruses cause mortality in ducks in an age-dependent manner. The underlying mechanisms for age-dependent pathogenicity in ducks are largely unknown. We studied the possible mechanism underlying the age-dependent pathogenicity of ducks infected with HP H5N6 avian influenza viruses using microarrays.

We showed that the infected ducks with HP H5N6 avian influenza viruses under 3 weeks old age show mortality, whereas the infected ducks over 4 weeks old age do not show any mortality. We intranasally infected Pekin ducks with 10^6^ EID_50_/_mL_ of A/Waterfowl/Korea/S57/2016 (clade 2.3.4.4.). Previous studies have shown the age-dependent mortality of Pekin ducks i.n. infected with HP H5N1 avian influenza viruses. When 2-week-old ducks were infected with 10^6^ EID_50_/_mL_ of HP avian influenza viruses, A/Vietnam/1203/04 (H5N1), A/ThailandPB/6231/04 (H5N1), and A/Egrett/HK/757.2/02 (H5N1), most of the infected ducks died. When 5-week-old ducks were infected with HP avian influenza viruses, A/ThailandPB/6231/04 (H5N1), and A/Egrett/HK/757.2/02, all the infected ducks had survived [36]. When 8-week-old Pekin ducks were i.n infected with 10^6^ EID_50_/_mL_ of HP avian influenza virus A/turkey/Turkey/1/05 (H5N1), all infected ducks died, while 12-week-old ducks infected with A/turkey/Turkey/1/05 (H5N1) did not show any mortality [37]. Five-week-old ducks (cherryvalley) experimentally i.n infected with 10^8^ EID_50_/_mL_ of H5N1 duck isolate in Japan did not die, but viruses were isolated from many organs, including the brain [38]. Age-dependent susceptibility of ducks to HP avian influenza virus was also observed during natural outbreaks of HP H5N1 avian influenza virus in commercial ducks in South Korea [39]. Two-week-old meat ducks infected with HP H5N1 virus showed severe morbidity and 12% of mortality with pathological lesions in the pancreas, liver, brain, and the heart. Meanwhile, the infected adult ducks on the breeder farm showed decreased egg production and feed consumption without mortality.

When we analyzed total gene expression in the lungs of infected two-week-old and four-week-old ducks by microarray and quantitative real-time PCR, most of the genes were equally expressed. Very few genes were differentially expressed between the lungs of 2- and 4-week-old ducks. Cell damage-related genes, CIDEA and ND2, and the immune response-related gene NR4A3 were significantly upregulated in the lungs of infected 2-week-old ducks compared to that in the lungs of infected four-week-old ducks. The previous study determined only innate immunity-related genes, IFN-γ, IL-6, RIG-1 (retinoic acid-inducible gene I), and IL-2 in the lungs of 2- and 5-week-old ducks infected with chicken/Hong Kong/220/97 (H5N1), A/Egret/HK/757.2/02 (H5N1), or Duck/Vietnam/218/05 (H5N1) using quantitative real-time PCR to find out the age-dependent susceptibility of ducks to HP H5N1 avian influenza viruses. It showed no significant expression of these genes in the lungs of infected ducks [39].

We used a chicken genome microarray for duck genome hybridization as no duck genome microarray is available yet. The previous study showed that duck genome samples could be hybridized to chicken gene microarrays as much as chicken genome samples can be hybridized to chicken gene microarrays [40]. The percentage that the chicken gene and duck gene samples were hybridized to the whole genome chicken microarray was 78 and 75, respectively. When a duck genome microarray becomes available, we may use it for the confirmation of these results.

In conclusion, age-dependent susceptibility to HP avian influenza virus in ducks may be due to differential expression of CIDEA and ND2. Further study may be needed to confirm whether these genes are important for controlling HP avian influenza viruses in ducks.

## Figures and Tables

**Table 1:** Duck primers for real-time PCR.

Primer Name	Sequence
ND2_775_F	GGCTTCATGCCAAAATGACT
ND2_972_R	GGGGGTGTTTAGGGTTTTGT
CIDEA_47_F	GTATCCGTGGGAGCATCTGT
CIDEA_270_R	GTGTCCACAACTGTGCCATC
SLC25A4_226_F	CAGATCACAGCGGAGAAACA
SLC25A4_377_R	TTGTCCTTGAAGGCGAAGTT
NR4A3_2658_F	GTCTTTTCTGGCGCTTTGTC
NR4A3_2849_R	ACTGCTGCTGGGATAGCATT
ACER3_6035_F	TGGCTACTGTGCAGAAATGC
ACER3_6218_R	TTGCTTGACTGGTGTGCTTC
GAPDH_F	TGTCTGGCAAAGTCCAAGTG
GAPDH_R	TCTCCATGGTGGTGAAGACA

## Supplementary Materials

The following are available online at https://www.mdpi.com/1999-4915/12/6/591/s1.
