# Supplementary material for: Age-Dependent Lethality in Ducks Caused by Highly Pathogenic H5N6 Avian Influenza Virus
Source: Viruses. 2020 May 29;12(6):591. doi: 10.3390/v12060591 (PMC7354466; doi:10.3390/v12060591)
Supplement: Supplementary file 1 [file viruses-12-00591-s001.pdf]

Supplementary Information

Table S1. Gene description

Gene Symbol	Public Gene ID	Description
ABCC9	XM_003640404	ATP-binding cassette, sub-family C (CFTR/MRP), member 9
ACER3	XM_004938943	alkaline ceramidase 3
AGTR2	XM_004940660	angiotensin II receptor, type 2
AKTIP	NM_001005838	AKT interacting protein
ALCAM	NM_205179	activated leukocyte cell adhesion molecule
ANXA1	NM_206906	annexin A1
ATCAY	XM_004948738	ataxia, cerebellar, Cayman type
ATP2B4	XM_004948440	ATPase, Ca++ transporting, plasma membrane 4
BCL6	NM_001012930	B-cell CLL/lymphoma 6
BLB3	XM_423039	Major histocompatibility complex class II beta chain BLB3, (similar to HLA class II, D beta chain)
BMP5	NM_205148	bone morphogenetic protein 5
BMP6	XM_418956	bone morphogenetic protein 6
C7	XM_424774	complement component 7
CARD11	NM_001006161	caspase recruitment domain family, member 11
CIDEA	NM_001195123	cell death-inducing DFFA-like effector a
CNR2	XM_004947898	cannabinoid receptor 2 (macrophage)
CXCL12	NM_204510	chemokine (C-X-C motif) ligand 12
CYBB	NM_001100286	cytochrome b-245, beta polypeptide
CYFIP2	XM_004944914	cytoplasmic FMR1 interacting protein 2
DAD1	NM_001007473	defender against cell death 1
DENND1B	XM_422194	DENN/MADD domain containing 1B
DLL1	NM_204973	delta-like 1 (Drosophila)

DOCK2	XM_425184	dedicator of cytokinesis 2
EGLN3	XM_004941757	egl-9 family hypoxia-inducible factor 3
ELF3	XM_419257	E74-like factor 3 (ets domain transcription factor, epithelial-specific)
ENPP2	NM_001198662	ectonucleotide pyrophosphatase/phosphodiesterase 2
EP300	XM_001233887	E1A binding protein p300
EPS8	XM_004937962	epidermal growth factor receptor pathway substrate 8
FAM49B	XM_004940034	family with sequence similarity 49, member B
FASLG	NM_001031559	Fas ligand (TNF superfamily, member 6)
FGFR2	NM_205319	fibroblast growth factor receptor 2
FOXO3	XM_001234495	forkhead box O3
GATA2	NM_001003797	GATA binding protein 2
GATA3	NM_001008444	GATA binding protein 3
GSN	NM_204934	gelsolin
GUCY1A2	XM_001233953	guanylate cyclase 1, soluble, alpha 2
HK1	NM_204101	hexokinase 1
HSP90AB1	NM_206959	heat shock protein 90kDa alpha (cytosolic), class B member 1
HSPD1	NM_001012916	heat shock 60kDa protein 1 (chaperonin)
IFNL3	NM_001128496	interleukin 28B (interferon, lambda 3)
IL13	NM_001007085	interleukin 13
JAG1	XM_415035	jagged 1
KCNC2	XM_001235254	potassium voltage-gated channel, Shaw-related subfamily, member 2
KLF2	XM_418264	Kruppel-like factor 2
LAMP1	NM_205283	lysosomal-associated membrane protein 1
LAMP3	NM_001146132	lysosomal-associated membrane protein 3
LCP1	NM_001008440	lymphocyte cytosolic protein 1 (L-plastin)

LFNG	ENSGALT00000006809	LFNG O-fucosylpeptide 3-beta-N-acetylglucosaminyltransferase
MOV10	ENSGALT00000002368	Mov10, Moloney leukemia virus 10, homolog (mouse)
MYC	NM_001030952	v-myc avian myelocytomatosis viral oncogene homolog
ND2	ENSGALT00000029098	NADH dehydrogenase subunit 2
NGF	NM_001293108	nerve growth factor (beta polypeptide)
NR1H4	NM_204113	nuclear receptor subfamily 1, group H, member 4
NR4A3	XM_419081	nuclear receptor subfamily 4, group A, member 3
PDE5A	XM_004941236	phosphodiesterase 5A, cGMP-specific
PLCG2	XM_004944257	phospholipase C, gamma 2 (phosphatidylinositol-specific)
POSTN	NM_001030541	periostin, osteoblast specific factor
PPARGC1A	NM_001006457	peroxisome proliferator-activated receptor gamma, coactivator 1 alpha
PPP1R13B	XM_004936376	protein phosphatase 1, regulatory subunit 13B
PRDX1	NM_001271932	peroxiredoxin 1
PTPN6	NM_001031484	protein tyrosine phosphatase, non-receptor type 6
RAB12	XM_003640800	RAB12, member RAS oncogene family
RAPGEF2	XM_004940909	Rap guanine nucleotide exchange factor (GEF) 2
ROBO2	XM_416674	roundabout, axon guidance receptor, homolog 2 (Drosophila)
SAMHD1	NM_001030845	SAM domain and HD domain 1
SEMA7A	NM_001199749	semaphorin 7A, GPI membrane anchor (John Milton Hagen blood group)
SGK1	NM_204476	serum/glucocorticoid regulated kinase 1
SGPL1	NM_001007946	sphingosine-1-phosphate lyase 1
SIK1	NM_204682	salt-inducible kinase 1
SLC25A4	NM_001006443	solute carrier family 25 (mitochondrial carrier; adenine nucleotide translocator), member 4
SLC6A4	NM_213572	solute carrier family 6 (neurotransmitter transporter), member 4
SMAD1	NM_001201455	SMAD family member 1

SMAD3	NM_204475	SMAD family member 3
SPHK1	XM_004946119	sphingosine kinase 1
STK3	NM_001031337	serine/threonine kinase 3
THRB	NM_001252221	thyroid hormone receptor, beta
TMEM117	XM_416041	transmembrane protein 117
TNFSF11	NM_001083361	tumor necrosis factor (ligand) superfamily, member 11
TOP2A	NM_204791	topoisomerase (DNA) II alpha 170kDa
TSPAN32	XM_004941440	tetraspanin 32
ZBTB20	XM_004938203	zinc finger and BTB domain containing 20
ZFPM1	XM_004944287	zinc finger protein, FOG family member 1

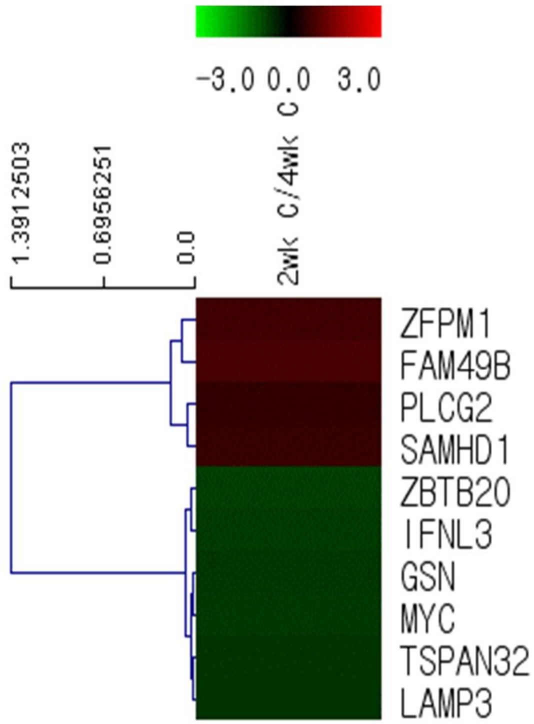

Fig. S1. Heat maps and fold change of anti-viral related genes in the lung of PBS-mock infected ducks: The differentially expressed genes in the lungs of 2-week-old duck compared to those in the lungs of 4-week-old duck.

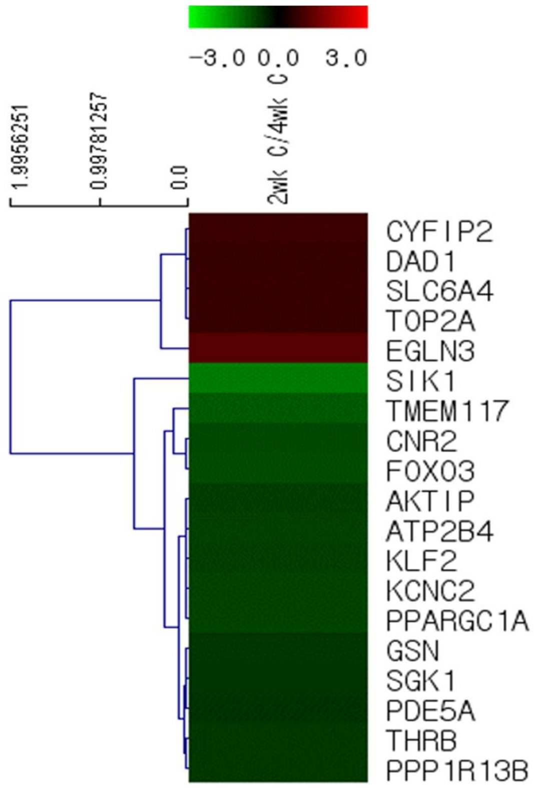

Gene title		Fold-change	p-value
Cell damage related gene	TMEM117	0.48	0.033
	SIK1	0.37	0.031

Fig. S2. Heat maps and fold change of cell-damage related genes in the lung of PBS-mock infected ducks: The differentially expressed genes in the lungs of 2-week-old duck compared to those in the lungs of 4-week-old duck.

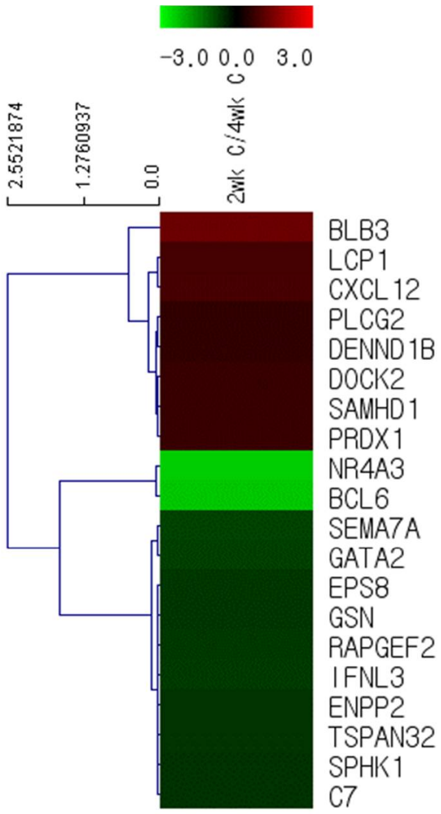

Gene title		Fold-change	p-value
Immune response related gene	BLB3	2.43	0.044
	NR4A3	0.19	0.048
	BCL6	0.20	0.024

Fig. S3. Heat maps and fold change of immune-response related genes in the lung of PBS-mock infected ducks: The differentially expressed genes in the lungs of 2-week-old duck compared to those in the lungs of 4-week-old duck.

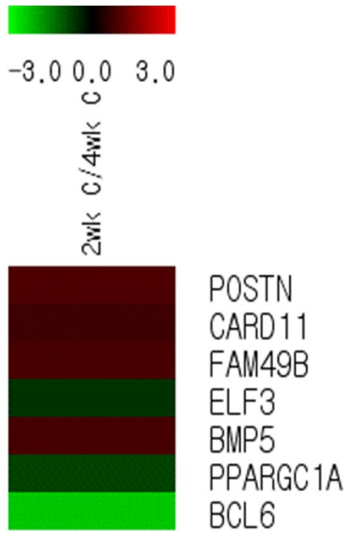

Gene title		fold change	p-value
Inflammatory response related gene	BCL6	0.20	0.024

Fig. S4. Heat maps and fold change of inflammatory-response related genes in the lung of PBS-mock infected ducks: The differentially expressed genes in the lungs of 2-week-old duck compared to those in the lungs of 4-week-old duck.

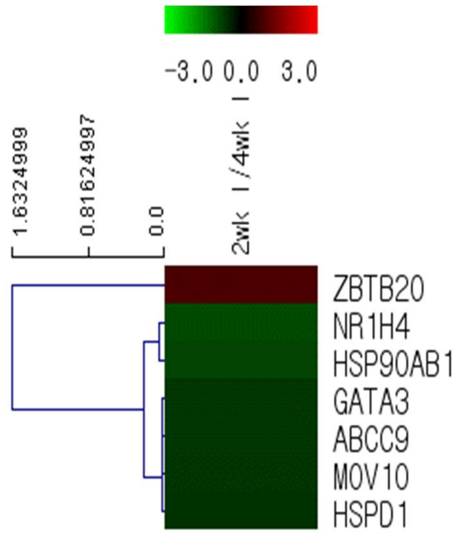

Fig. S5. Heat maps and fold change of anti-viral related genes in the lung of infected ducks: The differentially expressed genes in the lungs of 2-week-old infected duck compared to those in the lungs of 4-week-old infected duck.

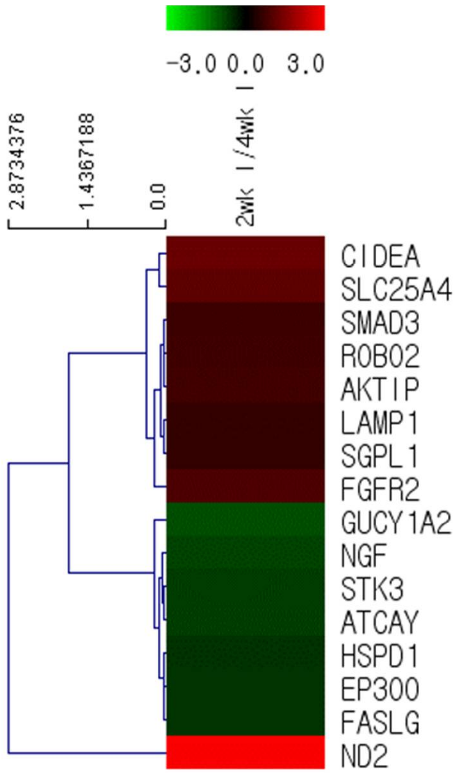

Gene title		fold change	p-value
Cell damage related genes	CIDEA	2.38	0.035
	SLC25A4	2.17	0.048
	ND2	7.84	0.046

Fig. S6. Heat maps and fold change of cell-damage related genes in the lung of infected ducks: The differentially expressed genes in the lungs of 2-week-old infected duck compared to those in the lungs of 4-week-old infected duck.

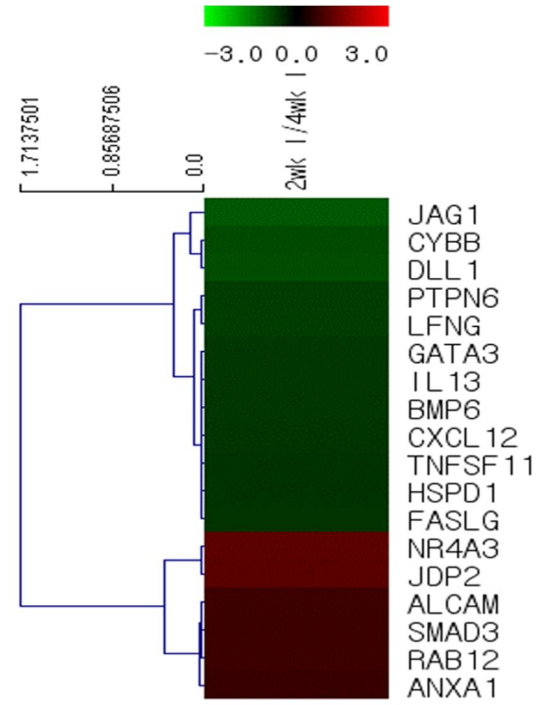

Gene title		fold change	p-value
Immune response related genes	NR4A3	2.14	0.056
	JAG1	0.49	0.058

Fig. S7. Heat maps and fold change of immune-response related genes in the lung of infected ducks: The differentially expressed genes in the lungs of 2-week-old infected duck compared to those in the lungs of 4-week-old infected duck.

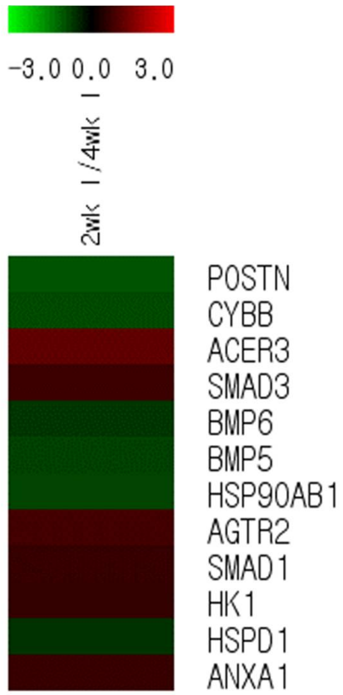

Gene title		fold change	p-value
Inflammatory response related genes	POSTN	0.50	0.045
	ACER3	2.22	0.044

Fig. S8. Heat maps and fold change of inflammatory-response related genes in the lung of infected ducks: The differentially expressed genes in the lungs of 2-week-old infected duck compared to those in the lungs of 4-week-old infected duck.

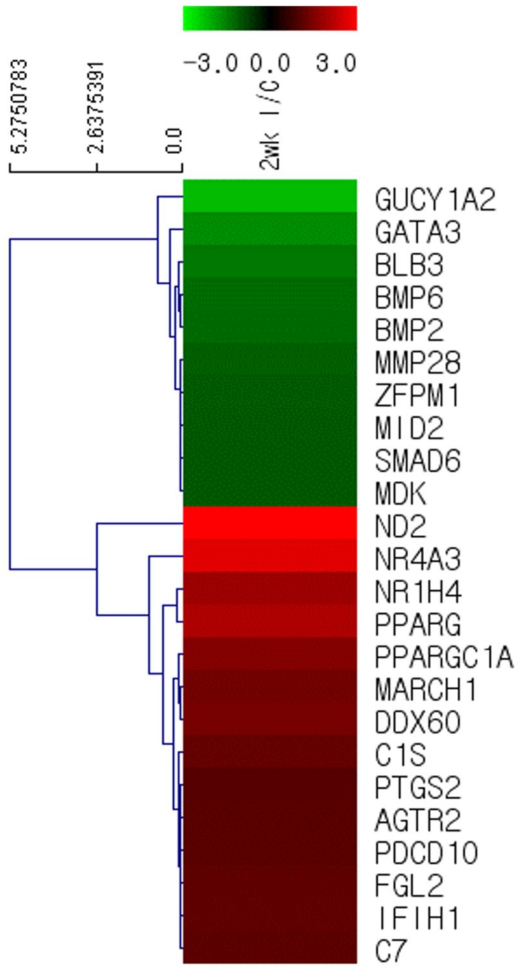

Gene title	fold change	p-value
GATA3	0.32	0.055
FGL2	2.19	0.034
NR1H4	3.63	0.048
C1S	2.31	0.047
GUCY1A2	0.22	0.027
SMAD6	0.49	0.038
ZFPM1	0.48	0.017
PPARG	4.14	0.043
BLB3	0.38	0.037
MMP28	0.46	0.045
BMP6	0.41	0.022
NR4A3	6.36	0.026
BMP2	0.42	0.044
PRDM1	2.45	0.043
AGTR2	2.07	0.039
PPARGC1A	2.97	0.03
MID2	0.49	0.045
MARCH1	2.57	0.011
DDX60	2.68	0.033
MDK	0.49	0.029
IFIH1	2.22	0.018
PTGS2	2.03	0.047
PDCD10	2.07	0.031
ND2	27.28	0.036
C7	2.14	0.027

Fig. S9. Heat maps and fold change of genes in the lung of 2-week-old infected ducks: The differentially expressed genes in the lung of 2-week-old infected duck compared to those in the lung of 2-week-old PBS-mock infected duck.

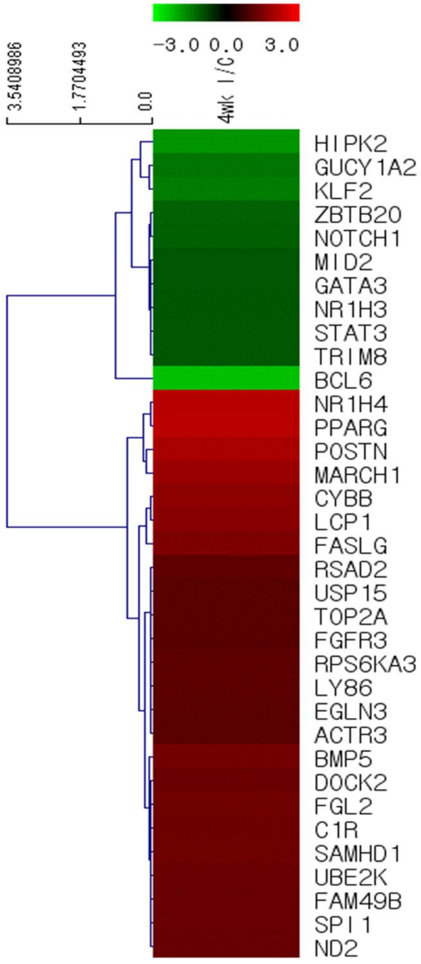

Gene title	fold change	p-value
GATA3	0.49	0.013
FGL2	2.50	0.057
USP15	2.08	0.044
NR1H4	4.50	0.025
HIPK2	0.29	0.043
ZBTB20	0.44	0.034
RPS6KA3	2.17	0.039
POSTN	4.00	0.028
GUCY1A2	0.38	0.033
C1R	2.46	0.046
CYBB	3.23	0.035
LCP1	3.10	0.017
PPARG	4.59	0.023
DOCK2	2.43	0.042
NOTCH1	0.46	0.041
LY86	2.16	0.036
FAM49B	2.38	0.054
SAMHD1	2.48	0.049
ACE	0.41	0.029
TOP2A	2.08	0.023
STAT3	0.48	0.036
KLF2	0.36	0.047
BMP5	2.60	0.038
RSAD2	2.23	0.021
MID2	0.50	0.029
MARCH1	3.58	0.04
UBE2K	2.39	0.019
FGFR3	2.07	0.047
SPI1	2.38	0.037
NR1H3	0.49	0.022
EGLN3	2.14	0.019
TRIM8	0.49	0.044
DPP4	2.55	0.025
ACTR3	2.13	0.031
FASLG	2.83	0.046
BCL6	0.21	0.042
ND2	2.36	0.015

Fig. S10. Heat maps and fold change of genes in the lung of 4-week-old infected ducks: The differentially expressed genes in the lung of 4-week-old infected duck compared to those in the lung of 4-week-old PBS-mock infected duck.
